# Characterization of the IS200/IS605 Insertion Sequence Family in *Halanaerobium Hydrogeniformans*

**DOI:** 10.3390/genes11050484

**Published:** 2020-04-29

**Authors:** Michael Sadler, Melanie R. Mormile, Ronald L. Frank

**Affiliations:** Department of Biological Sciences, Missouri University of Science and Technology, Rolla, MO 65409, USA

**Keywords:** transposable elements, mobile DNA, extremophilic bacteria, insertion sequences, IS200/605

## Abstract

Mobile DNA elements play a significant evolutionary role by promoting genome plasticity. Insertion sequences are the smallest prokaryotic transposable elements. They are highly diverse elements, and the ability to accurately identify, annotate, and infer the full genomic impact of insertion sequences is lacking. *Halanaerobium hydrogeniformans* is a haloalkaliphilic bacterium with an abnormally high number of insertion sequences. One family, IS200/IS605, showed several interesting features distinct from other elements in this genome. Twenty-three loci harbor elements of this family in varying stages of decay, from nearly intact to an ends-only sequence. The loci were characterized with respect to two divergent open reading frames (ORF), tnpA and tnpB, and left and right ends of the elements. The tnpB ORF contains two nearly identical insert sequences that suggest recombination between tnpB ORF is occurring. From these results, insertion sequence activity can be inferred, including transposition capability and element interaction.

## 1. Introduction

*Halanaerobium hydrogeniformans* is an anaerobic extremophile isolated in 2007 from Soap Lake, a meromictic haloalkaline lake in eastern Washington State. This organism gained attention for its unique metabolic capabilities and potential for industrial applications. Among the 2463 genes annotated in the genome, 72 transposase genes belonging to eight insertion sequence families were originally identified [1]. This put the genome at approximately 3% transposable elements, which is higher than most bacterial genomes [2].

Insertion sequences can be categorized into four groups based on their catalytic mechanisms for transposition. These groups are 1) the DDE, so called for the conserved catalytic DDE motif, 2) Y and 3) S for their tyrosine and serine residues at the catalytic site, and 4) HUH transposases that are further broken down into Y1 and Y2 transposase families (ISfinder: www-is.biotoul.fr). Transposases of the IS200/IS605 family of insertion sequences belong to the Y1 family of HUH transposases. Both IS200 and IS605 elements carry a transposase gene (tnpA), while IS605 members carry an additional gene (tnpB). These elements do not contain terminal inverted repeats, nor do they generate target site duplications upon insertion.

The TnpA protein functions as an obligatory dimer [3]. Each TnpA contains an HUH motif, a single catalytic tyrosine, and inserts the element 3’ to a specific tetra- or penta-nucleotide sequence [4]. For transposition to occur, each TnpA monomer binds an indispensable hairpin secondary structure present at each end of the element. In the well-characterized IS608 element of the IS200/IS605 family, the sequences of the left hairpin and right hairpin structures are the same [3]. The insertion site is identified through DNA–DNA interaction by a tetra-nucleotide guide sequence at the 5’ base of the hairpin structure [5].

The tnpB open reading frame (ORF), also known as OrfB, is approximately 1200 nucleotides in length and is dispensable for transposition. It has also been found associated with IS607, which carries a serine transposase [6]. OrfB is located in successive, divergent, or overlapping orientation with respect to tnpA [7]. Until recently, the function of TnpB was largely unknown. There is evidence to suggest that TnpB plays a role in transposition regulation of IS200 and IS605 elements [8].

Insertion sequences increase genetic diversity and genomic plasticity through genome rearrangements and the transfer of beneficial genes. However, insertion sequences are in general thought to be more damaging than beneficial and only provide a temporary selective advantage to their host [9]. As such, horizontal gene transfer is essential to the persistence of insertion sequences in the environment. It is hypothesized that insertion sequences undergo periodic invasion–expansion–extinction cycles [10,11]. These cycles are characterized through introduction to a new genome through horizontal gene transfer, expansion through replicative transposition, and extinction through various methods that eliminate or degrade insertion sequences beyond recognition in a genome [12].

This study presents a detailed characterization of the IS200/IS605 family members within *H. hydrogeniformans*. Six IS200/IS605 elements were originally annotated in the genome. After investigation, this number rose to 23 elements and 1 PATE-like (palindrome associated transposable element) sequence. Many of the IS200/IS605 elements were misidentified by insertion sequence annotation software, and exhibit unique disruptions and fragmentation not typically reported in insertion sequences. The phylogeny of these elements in comparison to their structural differences suggests recombination between elements is occurring.

## 2. Materials and Methods

### 2.1. Insertion Sequence Identification

The *Halanaerobium hydrogeniformans* genome sequence is recorded at the National Center for Biotechnology Information (NCBI), accession number CP002304.1. All genes annotated as insertion sequence, transposase, or integrase were used as query for a BLAST (Basic Local Alignment Search Tool) search against the Genbank database to determine potential products. The results were used as a query against the ISfinder [13] library to confirm insertion sequence identity. After confirmation, a representative ORF from each unique insertion sequence group was used for a BLAST search against the *H. hydrogeniformans* genome to reveal unidentified replicates. Insertion sequences in the genome were then identified with ISsaga [14] to ensure no insertion sequences were overlooked. ISsaga scans for insertion sequences in annotated genomes by comparing potential sequences against the ISfinder database. It then performs a BLASTN for replicons within the genome to identify partial elements or potential mobile elements not in the ISfinder library.

The surveyed elements were given loci numbers for organization and further reference. Loci numbers 1–23 were assigned to elements with increasing distance from the origin of replication. Element ends were identified by extending alignments to include sequences on either side of the ORF until regions of dissimilarity were observed. The ends were then used as a query for a BLAST against the genome, and matching sequences were verified to correspond to a previously identified insertion sequence. One PATE-like structure was found in this way.

### 2.2. Alignments

Alignment programs were used for pairwise and multiple sequence alignments. LALIGN [15] (version 36.3.7b, default parameters) was used to generate optimal local alignments between two sequences. Clustal Omega [16] (version 1.2.1, default parameters) was used to align multiple sequences.

### 2.3. Phylogenetic Analysis

To determine the relationship of the IS605 elements, phylogenetic analysis was conducted using NGPhylogeny.fr with nucleotide sequences from the tnpB ORFs. NGPhylogeny.fr is a free web service for phylogenetic analysis for non-specialists (http://www.ngphylogeny.fr/) [17,18]. Analysis was performed with default parameters using MAFFT (version 7.407_1) sequence alignment, BMGE (version 1.12_1) alignment curation, FastTree (version 2.1.10_1) tree inference, and Booster (version 0.2.4) branch support with a bootstrap of 100. Because the relationship between elements with the tnpB ORF inserts was of interest, the analysis was limited to three tnpB ORF types and excluded tnpB ORF with additional deletions. The resulting cladogram of the tnpB ORF is rooted with a similar sequence found in *Acetohalobium arabaticum* (Gen Bank CP002105.1) labeled as a pseudogene at locus ACEAR_1227.

### 2.4. Secondary Structure Identification

Regions of the element ends showing potential for hairpin formation were identified by aligning the element left and right end with its respective reverse complement. Regions showing significant alignment to their reverse complement were visually identified and subsequently examined with Mfold [19].

## 3. Results

### 3.1. Insertion Sequence Identification

In *Halanaerobium hydrogeniformans*, ISsaga identified 31 unique insertion sequences belonging to 16 IS families with a total of 108 elements. Of note were the IS200/IS605, IS607 family members. Manual curation of these elements revealed that they were a single IS200 member, 22 IS605 members, and one PATE-like sequence that was not identified by ISsaga.

Detailed characterization of insertion sequences in H. hydrogeniformans was limited to the IS200/IS605 family members. The elements are labeled loci 1–23 with increasing distance from the origin of replication. The locus numbers for each element, as well as some of the elements’ characteristics, which are further discussed in Section 3.2, Section 3.3, Section 3.4, Section 3.5, Section 3.6, Section 3.7 and Section 3.8 and are outlined in Table 1.

### 3.2. TnpA

**Figure 1:**
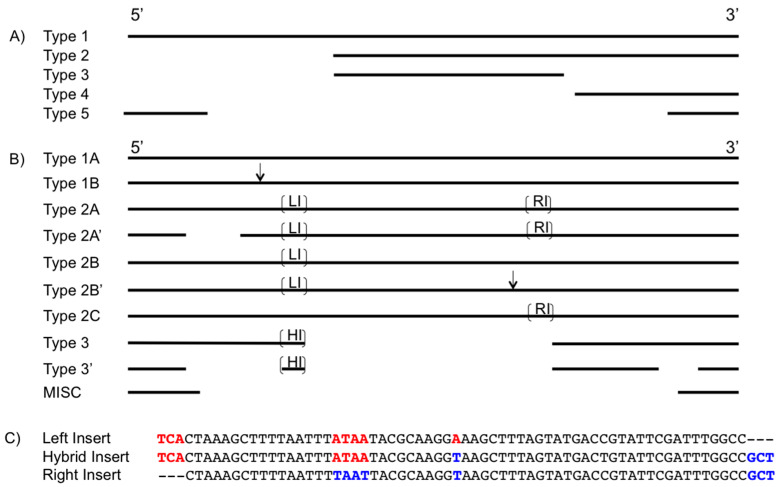
IS605 sequence visualization. Sequences are arranged from 5’ to 3’. Solid lines indicate nucleotide sequences and gaps indicate where the open reading frame (ORF) sequence is missing. Overlapping lines show where sequences are conserved between the different types. (**A**) IS605 tnpA ORF. (**B**) IS605 tnpB ORF. The down arrows of type 1B and type 2B’ show the relative position of a single nucleotide and putative IS21 insertion. The left insert (LI), right insert (RI), and hybrid insert (HI) are labeled with their abbreviation, placed in their relative location in the ORF, and shown with brackets. (**C**) IS605 tnpB ORF Inserts. Representatives of the left insert, right insert, and hybrid insert are shown in an alignment. Nucleotides common to the LI and HI are bolded in red text, while nucleotides common to the RI and HI are bolded in blue text.

Two tnpA ORF were identified in the genome: one belonging to an IS200 element (accession # ADQ14068.1) and the other to the IS605 elements (accession # WP_013405283.1). The IS200 tnpA had a single replicate, while there were 22 complete, partial, or fragmented copies of the IS605 tnpA ORF. Each potential protein contains a single Y1 Tnp superfamily domain. The translated sequences are 46% identical and 65% similar. Because the IS200 tnpA (locus 07) has a single replicate, and because IS200 does not produce target site duplications or have inverted repeats, it was not further characterized in this study. The 22 IS605 tnpA can be categorized into five types using structural differences in the ORF. These five types are shown in Figure 1a. Each of the 22 tnpA has a corresponding complete or partial divergent tnpB ORF.

Type 1 IS605 tnpA is a single replicate at locus 09 and is 405 nucleotides in length. This is the only IS605 tnpA that could produce a functional protein, as types 2–5 show significant degradation in the ORF.

Type 2 IS605 tnpA has two copies (loci 1 and 20). These ORF align with the 3’-most 234 nucleotides of type 1 and are missing 171 nucleotides from the 5’ end.

Type 3 IS605 tnpA has a single copy at locus 13. Type 3 ORF is missing 171 nucleotides from the 5’ end, 114 nucleotides from the 3’ end, and aligns with the central 120 nucleotides of type 1.

Type 4 IS605 tnpA has a single copy at locus 15 and aligns with the 3’-most 108 nucleotides of type 1.

Type 5 IS605 tnpA is the most commonly occurring IS605 tnpA type, with 17 copies. It is also the most fragmented of the five types. Unlike the others, type 5 IS605 tnpA does not annotate as a pseudo- or hypothetical gene by genomic annotation software. It is 122 nucleotides long, aligning with the 5’-most 63 nucleotides and the 3’-most 59 nucleotides of type 1 IS605 tnpA.

### 3.3. IS605 TnpB

There is a total of 22 IS605 tnpB open reading frames present in the genome (Accession #ADQ13737.1), each with a corresponding complete, partial, or fragmented divergent IS605 tnpA (see Table 1 for tnpA/tnpB pairings). These tnpB’s can be sorted into three primary groups with subgroups and one miscellaneous group. These groups are shown in Figure 1b.

Type 1 tnpB has four copies (loci 05, 08, 10, and 16). This ORF is 1254 nucleotides in length. This is not the most commonly occurring tnpB type but it is the ORF most likely to produce a functional protein, as the other types have various disruptions and deletions. The type 1A tnpB ORF encodes a protein containing three domains, a large orfB 605 superfamily domain, a 605 central region, and a terminal Zn-ribbon binding domain.

Type 1B tnpB has a single copy (locus 01) that aligns with the type 1A ORF, but contains a single nucleotide insertion at position 465.

Type 2A tnpB has three copies (loci 04, 06, and 09) and is 1382 nucleotides in length. These tnpB sequences align with the type 1A ORF with the exception of two additional 64 nucleotide inserts at position 433 (left insert, LI) and 1064 (right insert, RI). These inserts are further discussed below.

Type 2A’ tnpB has as a single copy (locus 13) and aligns with the type 2A ORF. It is classified as type 2A because it contains both LI and RI. It is denoted as a 2A* because it also is missing 173 nucleotides starting at nucleotide position 151.

Type 2B tnpB has three copies (loci 17, 19, and 20) and an ORF of 1318 nucleotides in length. This ORF aligns with type 2A tnpB but contains only the LI and no RI.

Type 2B’ tnpB has a single copy (locus 18) and has the same ORF and LI as other type 2Bs. This element is denoted separately from the other type 2B ORFs because it is disrupted by a putative IS21 element 2.6 kb in length.

Type 2C tnpB has two copies (loci 02, and 03) and is 1318 nucleotides in length. This tnpB aligns with the type 2A ORF with the exception that it contains only the RI and no LI.

Type 3 tnpB has five copies (loci 11, 12, 21, 22, and 23) and is 724 nucleotides in length. This element aligns with the type 2A ORF with the exception that it contains a hybrid insert (HI) at position 433 and is missing the 463 nucleotides that exist between the LI and RI of type 2A.

Type 3’ tnpB has a single copy (locus 15). This tnpB contains a HI like other type 3 tnpB ORFs but is in a more progressed state of deterioration. It is 499 nucleotides in length, and in addition to missing the region between inserts, it is lacking a 173-nucleotide segment beginning at position 146, and a 52 nucleotide sequence beginning at position 422.

A single miscellaneous (MISC) tnpB ORF (locus 14) was identified in the genome and is 172 nucleotides in length. This MISC tnpB ORF contains only the 5’-most 102 nucleotides, and the 3’-most 70 nucleotides of the type 1A ORF. Due to the lack of internal sequence or inserts, this element cannot be confidently placed in any other group.

### 3.4. IS605 TnpB Inserts

The tnpB ORF left insert (LI) and right insert (RI) mentioned previously can be sorted into three groups using their location within the ORF and the terminal three nucleotides on the 5’ and 3’ ends. The LI and RI are 64 nucleotides in length, while the hybrid insert (HI) is 67 nucleotides long. All inserts share a common 61-nucleotide central region, except where indicated in Figure 1c. The LI lacks a GCT sequence on the 3’ end and the RI insert lacks a TCA sequence on the 5’ end. The hybrid insert contains both the TCA and GCT sequences. This hybrid pattern persists internal to the insert ends between four mismatched nucleotides that are nine nucleotides apart.

### 3.5. IS605 TnpB Phylogeny

**Figure 2:**
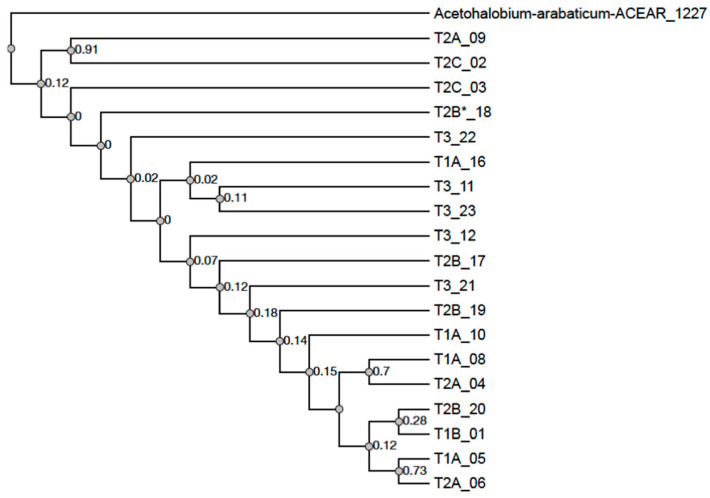
Cladogram of IS605 tnpB open reading frames. The cladogram is rooted to a similar sequence found in *Acetohalobium arabaticum*. Elements are labeled with their tnpB and locus position (example T2A_09 is a type 2A tnpB from locus 9). Analysis was performed with default parameters from NGphylogeny.fr using MAFFT (version 7.407_1) sequence alignment, BMGE (version 1.12_1) alignment curation, FastTree (version 2.1.10_1) tree inference, and Booster (version 0.2.4) branch support with a bootstrap of 100. Branch support values are low and sequences with known relationships (tnpB ORF inserts) do not clade.

A cladogram was constructed between the IS605 elements using the tnpB ORFs. Types 2A’, 3’, and MISC were excluded because of their more deteriorated state. The cladogram is shown in Figure 2 and is labeled with the tnpB type and locus (example, T2A_09, type 2A tnpB locus 9). Worth noting is that tnpB ORFs of the same type do not form a clade.

### 3.6. Element Ends

The left end (LE) of the element, defined here as the sequence downstream of the IS605 tnpA ORF, is 60 nucleotides in length for all elements except locus 13, which is missing 11 nucleotides closest to tnpA.

The right end (RE) of the element, defined here as the sequence downstream of the IS605 tnpB ORF, can be sorted into two types and one miscellaneous copy based on the presence of a 28-nucleotide insert. The type 1 RE, present in 16 of the 22 elements, is 132 nucleotides long. The type 2 RE, present in five elements (loci 01, 04, 06, 14, and 21) contains a 28-nucleotide insert at position 99 for a total length of 160 nucleotides. This 28-nucleotide insert does not show sequence similarity to the IS605 tnpB ORF inserts. The miscellaneous RE (locus 15) appears to be truncated and extends only 23 nucleotides past the 3’ end of its respective tnpB ORF. Unlike locus 18, where the remainder of the element can be clearly identified beyond the putative IS21 insertion, the remainder of the RE for locus 15 could not be located.

### 3.7. Hairpin Structures

**Figure 3:**
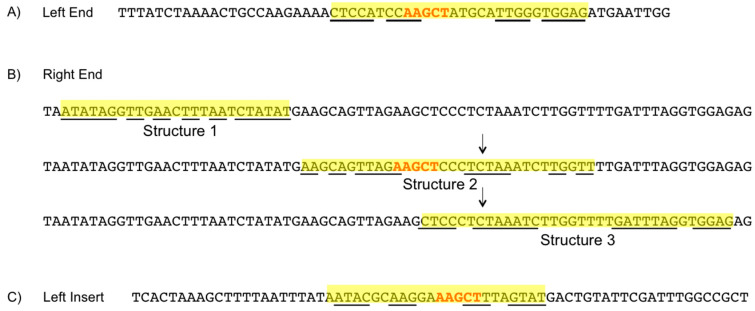
Sequences of IS605 potential hairpin structures from the tnpB strand organized from 5’ to 3’. Potential hairpin structures are highlighted in yellow. Complementary nucleotides forming the hairpin are underlined. Conserved AAGCT sequences between the hairpin loops are bolded in red text. (**A**) Left end. (**B**) Right end, where 50 nt from the 5’ end and 6 nt from the 3’ end are not shown. The arrows above structures two and three indicate where a 28-nt insert appears in elements with a type 2 RE disrupting these structures. (**C**) Left insert representative of the other LI, RI, and HI.

Both LE and RE sequences of the IS605 elements contain a hairpin structure required by IS200/IS605 family members for transposition. The LE has only 1 potential hairpin structure. It is composed of a 10-base-pair stem and an 8-nucleotide loop with ∆G of 6.31 kcal/mol. Figure 3a shows the LE sequence with the potential hairpin highlighted. The RE has three potentially competing structures, two of which are mutually exclusive. Structures 1, 2, and 3 form an imperfect stem with 8 out of 10, 9 out of 11, and 11 out of 13 base pairs with a 5, 7, and 8 nucleotide loop, and ∆G values of 1.87, 0.11, and 4.96 kcal/mol, respectively. The 28-nucleotide insert present in the type 2 RE disrupts structures 2 and 3, but not structure 1. Figure 3b shows the RE sequence with the three potential hairpin structures highlighted.

The inserts within the IS605 tnpB ORF (described in Section 3.4) also contain a hairpin structure. This structure is an imperfect stem with 7 out of 9 bp and a 5-nucleotide loop with a ∆G value of −1.54 kcal/mold. The potential stem-loop structure in the insert sequence is shown in Figure 3c.

### 3.8. PATE-Like Structure

**Figure 4:**
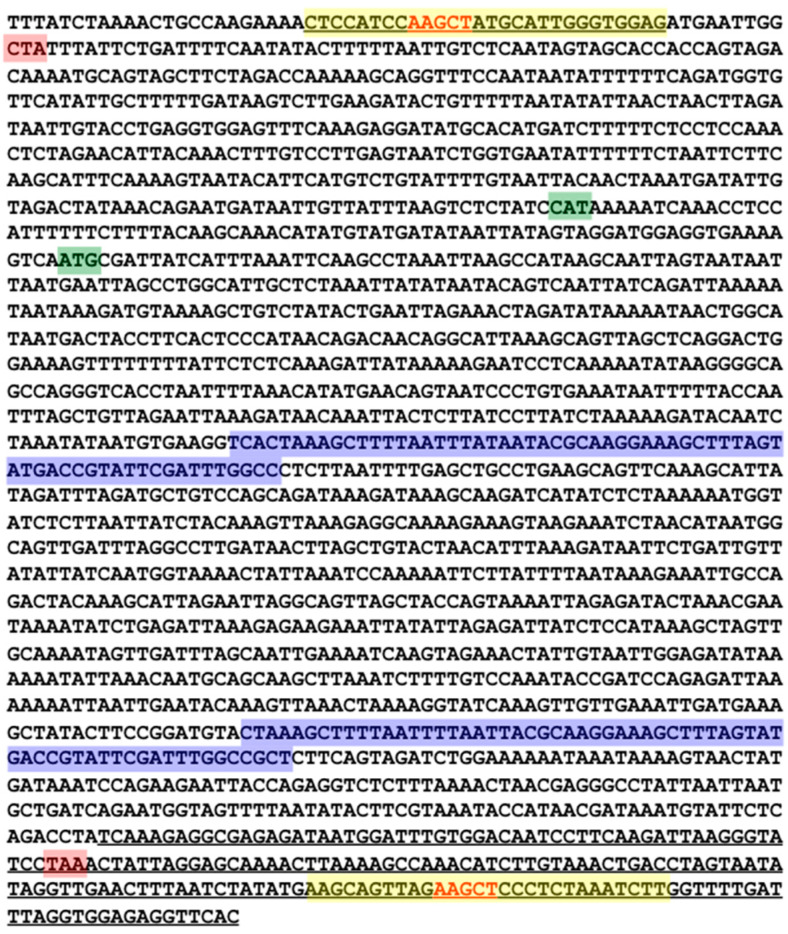
Locus 09 as a representative of a complete IS605 element in *H. hydrogeniformans*. The sequence shown is the tnpB coding strand and organized from 5’ to 3’. Start and stop codons are highlighted in green and red respectively. The tnpA and tnpB open reading frame (ORF) are in divergent orientation, with tnpA shown towards the 5’ end of the representative element. The tnpB ORF left and right inserts are highlighted blue. The left end and right end structure 2 stem-loop sequences are highlighted in yellow. An AAGCT pentanucleotide sequence in the loops of the left end structure and right end structure 2 are bolded in red text. The underlined sequences correspond to the sequence of the PATE-like element.

Palindrome-associated transposable elements are short elements harboring only the palindrome ends and are common to many insertion sequences. They are often transposable by a trans-acting transposase. One IS605 ends-only sequence was identified within the genome. This short sequence is approximately 271 nucleotides in length, beginning at nucleotide position 843,418 in the genome. This short sequence contains (left to right) 28 nucleotides of the LE, no sequence of the IS605 tnpA ORF, the last 58 nucleotides of tnpB ORF, and the entire 160 nucleotides of a type 2 RE. The 28 nucleotides of the LE contain the predicted hairpin structure. The PATE-like element is shown underlined in Figure 4.

## 4. Discussion

### 4.1. Insertion Sequence Identification

IS605 elements in *Halanaerobium hydrogeniformans* were chosen for detailed characterization because of their progressed stages of decay and because IS200/IS605 elements do not have a strong preference for cis transposition, the preference for a transposase to act on the element from which it was transcribed. Many of the IS605 elements were initially identified as pseudogenes, IS1341, and IS607 elements, because partial IS605 tnpA sequences were not detected. Additionally, the most closely related tnpB in the ISfinder library was a tnpB of an IS607 which carries a serine transposase. The misidentification of many of the IS605 elements in *H. hydrogeniformans* by automated methods highlights the importance of manual identification and the need for contributions to improve insertion sequence libraries.

### 4.2. IS605 TnpB Phylogeny

The relatedness of the tnpB ORF can be seen through the structural similarities (tnpB ORF inserts) and it was expected that elements sharing inserts (LI/RI/HI) would form a clade on the cladogram. However, as seen in Figure 2, tnpB ORFs that share inserts do not clade. Failure of structurally similar ORFs to form clades and poor branch support between elements with clear relatedness suggest these sequences have been shuffled through recombination. However, there is limited support for recombination between insertion sequences of an obligate endosymbiont [20]. Recombination of insertion sequences could play an important role in invasion–expansion–extinction cycles by spreading deleterious segments or through gene conversion.

### 4.3. Type 5 TnpA

It is worth noting that the most commonly occurring IS605 tnpA is type 5 (17 of the 22 elements). The abundance of IS605 elements containing a type 5 tnpA may be a result of increased rates of transposition relative to the other IS605 tnpA types. Two mechanisms could account for an increased rate of transposition; either size reduction increases transposition frequency, or the missing tnpA nucleotide sequence could have a regulatory function in addition to encoding the TnpA protein. IS605 exclusively excises from, and preferentially inserts into, ssDNA [21]. This preference leads to a bias towards lagging strand template insertion when transposition is coupled with host replication. As element size increases, the probability that both ends of the element exist as ssDNA decreases. Thus, as element size decreases, there is an increase in genome-replication-associated transposition events [22]. The 282 nucleotide size reduction of an element with a type 5 tnpA may increase the frequency of transposition by increasing the time spent in a ssDNA state during replication. This explanation relies on genome-replication-associated transposition and a bias for lagging strand insertion. While it could be a result of inversion events or misassembly, there is no apparent preference for leading or lagging strand insertion (10 of 22 tnpA on leading strand), as seen in Table 1.

The TnpB protein serves as a potential IS605 transposition regulatory protein and has been shown to inhibit IS605 excision and insertion. It is hypothesized that TnpB inhibits transposition by binding the terminal DNA hairpin structures or the TnpA protein itself [8]. It is possible that the TnpB protein binds ssDNA of the IS605 tnpA ORF sequence, inhibiting TnpA binding or dimerization and subsequently preventing transposition. If the region of binding were missing (Figure 1a, Type 5) TnpB could not inhibit transposition, and elements without this sequence would experience an increased rate of transposition. Alternatively, the disproportional number of type 5 tnpA may be a relic of early formation after insertion sequence acquisition and selective pressure against functional TnpA proteins.

### 4.4. IS605 TnpB Inserts

The left insert (LI) and right insert (RI) of the tnpB ORF show high sequence similarity, indicating that they originated from the same source. Neither the insert nor any part of it is found in the genome outside a tnpB ORF. The LI and RI differ by their 5’ and 3’-most three nucleotides. The differentiating nucleotide sequence at either end suggests an imprecise excision of the insert before insertion into the IS605 tnpB ORF. All LI contain a TCA as the 5’-most three nucleotides, while all RI contain a GCT as the 3’-most three nucleotides. The hybrid insert (HI) is 67 nucleotides in length and contains both TCA and GCT nucleotides at the 5’ and 3’ ends respectively (Figure 1c). This pattern indicates that a recombination event has occurred between a LI and a RI to form a HI. This same hybrid insert pattern persists internal to the insert ends. The LI contains an ATAA and an A at nucleotide positions 20 and 33 respectively, while the RI contains a TAAT and T at these positions. The HI contains the ATAA from the LI and T from the RI. This suggests that the initiating endonuclease for recombination between these inserts has an affinity for the sequence between positions 20 and 33 of the insert.

All observed elements with a HI were missing the inter-insert sequence. Because no elements with duplicated inter-insert sequences were identified in the genome, it is proposed that these HI were formed from the recombination of a LI and RI of the same element.

The independent insertion of all the LI and RI to the same relative location within the tnpB ORF is unlikely. Their reoccurrence in tnpB ORF is thus likely a result of two insertion events and the replication of those elements. As such, it is reasonable to expect that the presence of these inserts in the tnpB ORF does not impede transposition of the IS605 elements.

### 4.5. Element Ends

Element ends of IS200/IS605 family members contain hairpin structures indispensable for transposition. In the IS608 elements, the left end (LE) and right end (RE) structures have the same sequence [3,5], although this is not always the case for IS200/IS605 elements. The LE sequence for all IS605 elements in *H. hydrogeniformans* is highly conserved and extends 60 nucleotides downstream of the tnpA ORF. The LE has the potential to form a single hairpin structure (Figure 3a) but shows no significant sequence similarity to the RE. The RE of the IS605 elements is 132 nucleotides in length and has the potential to form three hairpin structures (Figure 3b). Structures 2 and 3 have significant overlap, structures 1 and 2 are separated by a single nucleotide, and structures 1 and 3 are separated by 14 nucleotides. It seems likely that these structures would compete and may impede transposition.

There has previously been speculation that the terminal hairpin structures of IS200/IS605 elements serve as a transcriptional terminator as well as preventing ribosome binding. It has since been established that they play a mechanistic role in transposition. Potentially competing structures may further serve a regulatory role by preventing the required hairpin structure from being bound by a TnpA monomer. Competitive structures have been reported before, although in these instances it was clear which structures were required for transposition, as only a single common structure was observed between the LE and the RE [7].

There is a conserved penta-nucleotide sequence (AAGCT) in the loop of both the LE structure and RE structure 2. This sequence appears in bolded red text within the highlighted hairpin structures (Figure 3a,b). Because the RE and LE hairpins differ so drastically in sequence, the conservation of this pentanucleotide sequence suggests it is important for hairpin recognition by TnpA. If RE structure 2 is the functional hairpin, it is unclear to what degree the RE structures 1 and 3 would affect transposition. Of note is a hairpin structure in the tnpB ORF insert (Figure 3c); this hairpin contains an AAGCT penta-nucleotide sequence in the loop.

At five loci (01, 04, 06, 14, and 21) a 28-nucleotide long sequence has inserted into the RE. This insert occurs immediately after nucleotide 21 of RE structure 2 and nucleotide 8 of RE structure 3 disrupting both structures. Because elements containing this RE insert have replicated it is unclear what impact this insert has on transposition.

## 5. Conclusions

Although only a single element contains an intact IS605 tnpA, all IS605 elements reported here contain intact element ends and are likely capable of transposition by a TnpA acting in trans. The RE and LE structure sequences suggest that hairpin recognition is dependent on a conserved penta-nucleotide sequence present in the hairpin loop. The inserts in the tnpB ORF provide structural differences that can be used to infer recombination between insertion sequences. This detailed survey of IS200/IS605 elements and fragments provides a snapshot of how insertion sequences transpose, interact, and degrade within bacterial genomes. Understanding these processes is important, as insertion sequences and their fragments influence host genomes through gene regulation, horizontal gene transfer, and increasing genetic plasticity.

## Figures and Tables

**Table 1:** Characteristics of IS200 and IS605 elements.

Locus	TnpA Type	TnpB Type	LE	RE	TnpA Locus ID	TnpB Locus ID	Leading/Lagging
1	Type 2	1B	consensus	type 2	HALSA_RS01255	HALSA_RS01260	Lead
2	Type 5	2C	consensus	type 1	N/A	HALSA_RS01330	Lead
3	Type 5	2C	consensus	type 1	N/A	HALSA_RS01515	Lag
4	Type 5	2A	consensus	type 2	N/A	HALSA_RS01645	Lead
5	Type 5	1A	consensus	type 1	HALSA_RS02280	HALSA_RS02275	Lead
6	Type 5	2A	consensus	type 2	HALSA_RS02590	HALSA_RS02585	Lag
7	IS200	N/A	unknown	unknown	HALSA_RS03110	N/A	Lag
8	Type 5	1A	consensus	type 1	N/A	HALSA_RS03165	Lag
9	Type 1	2A	consensus	type 1	HALSA_RS03745	HALSA_RS03750	Lag
10	Type 5	1A	consensus	type 1	N/A	HALSA_RS04080	Lag
11	Type 5	3	consensus	type 1	N/A	HALSA_RS12615	Lag
12	Type 5	3	consensus	type 1	N/A	HALSA_RS12630	Lag
13	Type 3	2A’	consensus	type 1	HALSA_RS12635	HALSA_RS05500	Lag
14	Type 5	MISC	consensus	type 2	N/A	N/A	Lead
15	Type 4	3’	consensus	MISC	HALSA_RS12645	HALSA_RS12715	Lead
16	Type 5	1A	consensus	type 1	N/A	HALSA_RS06215	Lag
17	Type 5	2B	consensus	type 1	N/A	HALSA_RS07530	Lag
18	Type 5	2B’	consensus	type 1	N/A	HALSA_RS08275	Lead
19	Type 5	2B	consensus	type 1	N/A	HALSA_RS08865	Lag
20	Type 2	2B	consensus	type 1	HALSA_RS11165	HALSA_RS11170	Lead
21	Type 5	3	consensus	type 2	N/A	HALSA_RS12685	Lead
22	Type 5	3	consensus	type 1	N/A	HALSA_RS12690	Lead
23	Type 5	3	consensus	type 1	N/A	HALSA_RS12700	Lag
PATE	NA	NA	Hairpin	type 2	N/A	N/A	Lag
